# Video-Assisted Thoracoscopic Surgery in Patients With Clinically Resectable Lung Tumors

**DOI:** 10.1155/DTE.2.151

**Published:** 1996

**Authors:** H. Sakai, P. J. Borgstein, M. A. Cuesta, S. Meijer, J. C. van Mourik, G. Sutedja, P. E. Postmus

**Affiliations:** 1 European Cancer Centre Amsterdam The Netherlands; 2 Department of Surgery Free University Hospital Amsterdam The Netherlands; 3 Department of Pulmonology Free University Hospital Amsterdam The Netherlands; 4 Department of Surgery Tokyo Medical College 6-7-1 Nishishinjuku, Shinjuku-ku Tokyo 160 Japan

## Abstract

To investigate the feasibility of thoracoscopic resection, a pilot study was performed in patients with clinically resectable lung tumors. In 40 patients, Video-assisted thoracic surgery (VATS) was performed because of suspicion of malignancy. There were 29 men and 11 women with a median age of 54.8 years (range 18 to 78). Preoperative indications were suspected lung cancer and tumor in 27 patients, assessment of tumor resectability in 7 patients, and probability of metastatic tumors in 6 patients. The final diagnoses in the 27 patients with suspected lung cancer were 12 primary lung cancers, 6 lung metastases, and 9 benign lesions. The success rates for VATS (no conversion to thoracotomy) were 1 of 12 (8.3%) for resectable stage I lung cancer, 8 of 12 (66.7%) for metastatic tumors, and 9 of 9 (100%) for benign tumors. With VATS, 6 of 7 patients (85.7%), possible stage III non-small cell lung cancer, an explorative thoracotomy with was avoided, significantly reducing morbidity. The reasons for conversion to thoracotomy were 1) oncological (N2 lymph node dissection and prevention of tumor spillage) and 2) technical (inability to locate the nodule, central localization, no anatomical fissure, or poor lung function requiring full lung ventilation). The ultimate diagnoses were 19 lung cancers, 12 metastatic lung tumors, and 9 benign lung tumors. Our data show the limitations of VATS for malignant tumors in general use. These findings, together with the fact that experience in performing thoracoscopic procedures demonstrates a learning curve, may limit the use of thoracoscopic resection as a routine surgical procedure, especially when strict oncological rules are respected.


*Diagnostic and Therapeutic Endoscopy*, 1996, Vol. 2, pp. 151–155
Reprints available directly from the publisher
Photocopying permitted by license only

© 1996 OPA (Overseas Publishers Association)
Amsterdam B.V. Published in The Netherlands
by Harwood Academic Publishers GmbH
Printed in Singapore

### Video-Assisted Thoracoscopic Surgery in Patients with Clinically Resectable Lung Tumors

H. SAKAI, P. J. BORGSTEIN, M. A. CUESTA, S. MEIJER, J. C. VAN MOURIK, G. SUTEDJA, and P. E. POSTMUS

*European Cancer Centre, Amsterdam, the Netherlands (H.S.); Departments of Surgery (P.J.B., M.A.C., S.M., J.C.V.M.), Pulmonology (G.S., P.E.P.), Free University Hospital, Amsterdam, the Netherlands; and Department of Surgery, Tokyo Medical College, Tokyo, Japan (H.S.)*

*(Received February 24, 1995, in final form September 5, 1995)*

To investigate the feasibility of thoracoscopic resection, a pilot study was performed in patients with clinically resectable lung tumors. In 40 patients, Video-assisted thoracic surgery (VATS) was performed because of suspicion of malignancy. There were 29 men and 11 women with a median age of 54.8 years (range 18 to 78). Preoperative indications were suspected lung cancer and tumor in 27 patients, assessment of tumor resectability in 7 patients, and probability of metastatic tumors in 6 patients. The final diagnoses in the 27 patients with suspected lung cancer were 12 primary lung cancers, 6 lung metastases, and 9 benign lesions. The success rates for VATS (no conversion to thoracotomy) were 1 of 12 (8.3%) for resectable stage I lung cancer, 8 of 12 (66.7%) for metastatic tumors, and 9 of 9 (100%) for benign tumors. With VATS, 6 of 7 patients (85.7%), possible stage III non-small cell lung cancer, an explorative thoracotomy with was avoided, significantly reducing morbidity. The reasons for conversion to thoracotomy were 1) oncological (N2 lymph node dissection and prevention of tumor spillage) and 2) technical (inability to locate the nodule, central localization, no anatomical fissure, or poor lung function requiring full lung ventilation). The ultimate diagnoses were 19 lung cancers, 12 metastatic lung tumors, and 9 benign lung tumors. Our data show the limitations of VATS for malignant tumors in general use. These findings, together with the fact that experience in performing thoracoscopic procedures demonstrates a learning curve, may limit the use of thoracoscopic resection as a routine surgical procedure, especially when strict oncological rules are respected.

**KEY WORDS:** Lobectomy, lung cancer, thoracoscopy, video-assisted thoracic surgery (VATS)

### INTRODUCTION

Thoracoscopy has been performed by pulmonologists and thoracic surgeons for more than 80 years.[1] In 1910 the first thoracoscopy was performed by H. C. Jacobaeus, using a modified cystoscope.[1] Initially, thoracoscopy was used for diagnostic purposes in the parietal pleura, diaphragm, pericardium, mediastinum, and most of the lung.[2,3] Then, therapeutic applications, such as talc poudrage, for a spontaneous pneumothorax became possible. But interest in thoracoscopy decreased due to the development of safer operative techniques and the introduction of antituberculous drugs.

In the 1970s, new interest in thoracoscopy emerged due to the development of video imaging technology and new endoscopic instruments. In 1980 Boutin organized the First International Symposium on Thoracoscopy.[4] Many thoracic surgeons have since extended the applications of this procedure to include therapeutic interventions such as mechanical pleurodesis, pleurectomy, bullectomy for pneumothorax, wedge resection, lobectomy and mediastinal mass exploration, and resection for benign and malignant tumors.[2–13] Even pneumonectomy has been reported to be feasible.[14,15]

For resectable lung tumors, surgery is the first treatment of choice. However, the morbidity for thoracotomy is considerable.[16] Thoracoscopic lobectomy may improve postoperative recovery and reduce the period of hospitalization, which may decrease health care costs.

At present, many surgeons are interested in thoracoscopic lobectomy for lung cancer, but its efficacy has not been clearly demonstrated.[5–9,14,15,17–23] Two major issues are important: the risk of inadequate nodal staging, re-

Address for correspondence: Harumasa Sakai, M.D., Ph.D., Department of Surgery, Tokyo Medical College, 6-7-1 Nishishinjuku, Shinjuku-ku, Tokyo 160, Japan sulting in residual tumor, and possible incomplete resection and tumor spillage.[24]

To investigate the feasibility of thoracoscopic lobectomy, a pilot study was performed using video-assisted thoracoscopic surgery (VATS) in 40 patients with clinically resectable lung tumors, with the use of anterior utility thoracotomy.

### MATERIALS AND METHODS

### Patients

Forty patients with suspected malignant lung tumors were entered into this study. There were 29 men and 11 women, and the median age was 54.8 years (range 18 to 78). Preoperative diagnoses were tumors suspected to be malignant in 27 patients and probable metastatic lung lesions in 6 patients; in another 7 patients with advanced lung cancer, thoracoscopy was used to assess the resectability of the tumor (Fig. 1).

### Pretreatment Investigation

The standard work-up procedure for clinical staging of lung cancer was performed including chest X-rays, computed tomographic scans of the lung and mediastinum, bronchoscopy, and lung function studies.

### Surgical Procedure

VATS was performed under general anesthesia with a double lumen tube to collapse the ipsilateral lung. The patient was placed in the lateral position to prepare for a possible thoracotomy. In most patients, three trocars (sixth intercostal middle axillary line, fourth intercostal anterior, and posterior axillary lines) were introduced to examine, dissect, and finally resect the tumor. An anterior utility thoracotomy (3 to 10 cm) was performed to aid the thoracoscopic procedure for dissection and removal of the resected specimens when necessary.

### RESULTS

Resection of four of six lung tumors suspected of being metastatic was performed thoracoscopically. In the other two patients, a conversion to thoracotomy was necessary (Fig. 1).

In 27 patients with probable primary lung tumors, resection of the lesion was performed by VATS in 14. In 13 patients a conversion to thoracotomy was necessary. Final diagnoses in these patients were 12 primary lung cancers, 6 lung metastases, and 9 benign lesions.

All conversions to a thoracotomy were in patients with malignant lesions; 11 with primary lung tumors and 2 with lung metastases. This means that in only 1 of 12 patients with lung cancer was VATS possible. This patient had stage I disease. The other 11 patients had stage I ($n$ = 7), stage II ($n$ = 1), and stage IIIA ($n$ = 3) disease. The reasons for conversion both for patients with lung metastasis (4 of 12) and lung cancer (11 of 12) are described in Table 1. According to our data, conversion to thoracotomy occurred in 7 of 11 patients due to technical difficulties (no anatomical fissure in 1, impossible to find the nodule and/or centrally located in 5, and poor lung function requiring whole lung ventilation in 1) (Table 1). In another 4 patients conversion to thoracotomy occurred due to definitive N2 positive in 1 and to prevent tumor spill in 3 (Table 1).

The success rates for VATS were 1 of 12 (8.3%) for lung cancers, 8 of 12 (66.7%) for metastatic lung tumors, and 9 of 9 (100%) for benign lung tumors.

With VATS, in 6 of 7 (85.7%) patients with possible stage III non-small cell lung cancer, an explorative thoracotomy was avoided. Reasons for inoperability were N2 positive tumor. (3 patients), N3 positive tumor (1 patient), and 14 tumor (2 patients) (Fig. 2).

The overall duration of thoracoscopic lobectomy was 3.5 hours. The duration for metastatectomy was 1.8 hours and resection of benign lung tumors was 1.3 hours.

### DISCUSSION

Twelve of 40 patients in whom VATS was performed had clinically resectable non-small cell lung cancers. We could perform thoracoscopic lobectomy in only 1 patients (1 of 12 = 8.3%). Hazelrigg et al.[22] reported in their VATS study group data that in 439 patients (439 of 1820 = 24.1%) eventual conversion to a thoracotomy occurred. Video-assisted lobectomy was reported in 38 patients and 91 patients underwent lobectomy by thoracotomy after a VATS procedure.[22] The success rate for thoracoscopic lobectomy was therefore 38/(38 + 91 = 29.5%). But it is unclear in how many patients conversion to wedge resection or pneumonectomy occurred or how many tumors turned out to be inoperable. The success rate for thoracoscopic lobectomy must therefore be less than 29.5%.

Hazelrigg et al.[22] also described their inability to find the lung tumor as a frustrating problem. This occurred in 7.5% of patients, resulting in thoracotomy. In our pilot study, this was the reason for conversion in 4 of 33 (12%) patients. In another 3 patients conversion to thoracotomy was due to technical difficulties (no anatomical fissure, centrally located nodule, or poor lung function requiring whole lung ventilation in 1 patient) (Table 1). Many tumor

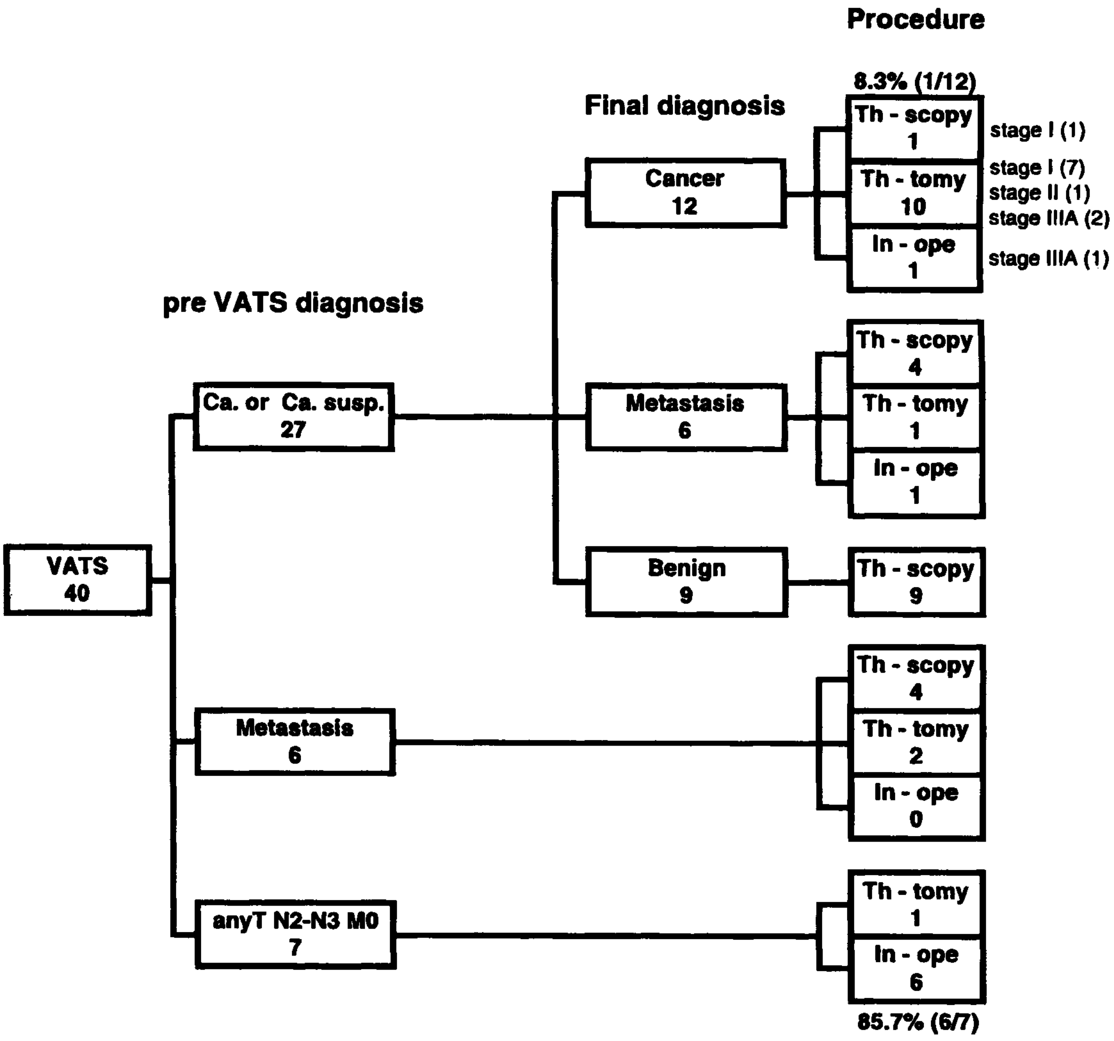

**Figure 1** VATS in patients with clinically resectable lung tumors. In 40 patients, VATS were performed for suspected malignant lung tumors. The success rate for VATS (no conversion to thoracotomy) in clinically resectable non-small cell lung cancers was 8.3%. VATS prevented explorative thoracotomies in 85.7% of the patients, significantly reducing morbidity.

**Table 1** Reasons for Conversion to Thoracotomy

| | *NSCLC* | *MLT* | *BLT* | *Total* |
|---|---|---|---|---|
| N2 positive | 1 | | | 1 |
| Prevent tumor spill | 3 | 4 | | 7 |
| Nodule not found | 4 | | | 4 |
| Centrally located | 1 | | | 1 |
| No fissure | 1 | | | 1 |
| Poor lung function | 1 | | | 1 |
| VATS | 1 | 8 | 9 | 18 |
| Total | 12 | 12 | 9 | 33 |

NSCLC, non-small cell lung cancer; MLT, metastatic lung tumor; BLT, benign lung tumor.

localization techniques have been published in which wire localization and endoscopic ultrasound seemed to be somewhat useful[25,26] (Fig. 3). It is very important, however, to apply oncological principles in thoracoscopic resection of malignancies.

Our data clearly show the limitations of VATS for malignant tumors in general. This is in contrast with the more optimistic results of proponents of thoracoscopic resection as previously reported, in which patient selection may influence the success rate of the thoracoscopic approach.

VATS did prevent explorative thoracotomies in 85.7% of the patients, significantly reducing morbidity. Therefore, thoracosopic resection of malignancies is expected to be of only limited usefulness in patients with clinically resectable malignant pulmonary nodules.

These findings together with the fact that thoracoscopic procedures demonstrate a learning curve may limit their use as routine surgical procedures for lobectomy in the

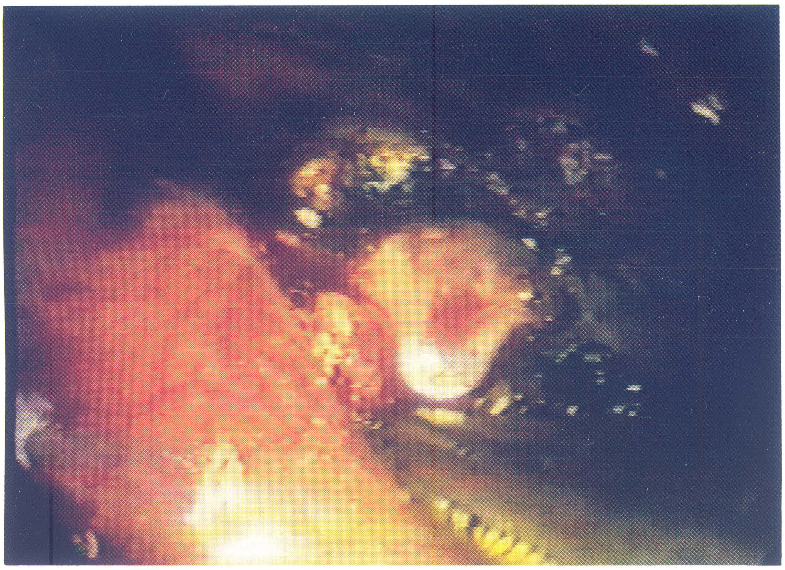

**Figure 2** With VATS, we diagnosed N2 positivity, which was not diagnosed before operation. An explorative thoracotomy was avoided in 85.7% of patients.

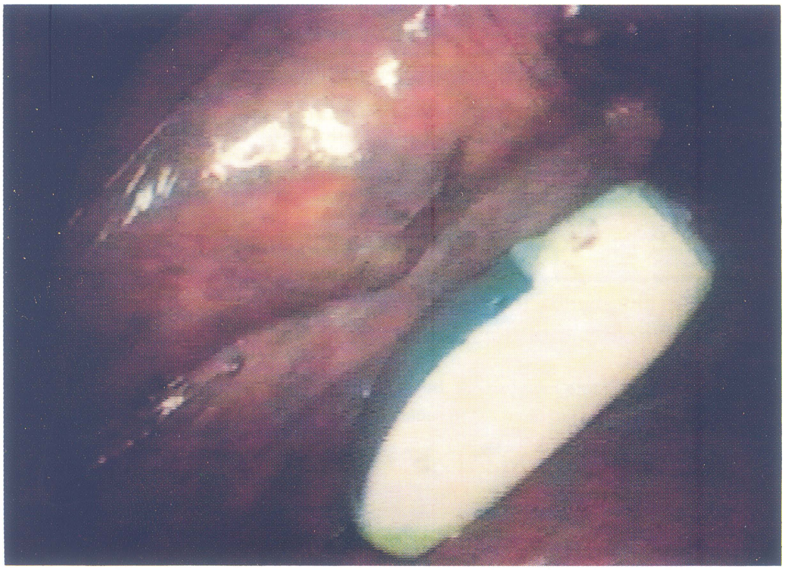

**Figure 3** Many tumor localization techniques have been published. We tried to localize the tumor with endoscopic ultrasound.

suspicion of a malignancy, especially if strict oncological rules are followed.

### ACKNOWLEDGEMENTS

This European Cancer Centre fellowship is supported by Nationale Nederlanden.

Dr. Sakai thanks Professor Harubumi Kato, (Department of Surgery, Tokyo Medical College) for giving him the chance to work in the European Cancer Centre.

### REFERENCES

1. Braimbridge MV. The history of thoracoscopic surgery. Ann Thorac Surg 1993;56:610–614.
2. Colt HG. Thoracoscopy: new frontiers. Am Coll Chest Physicians Pulmonary Perspect 1992;9:1–4.
3. Mathur P, Martin W. Clinical utility of thoracoscopy [editorial]. Chest 1992;102:2–4.
4. Boutin C, Viallat JR, Cargnino P, Farisse P. Current indications for thoracoscopy. Rev Fr Mal Respir 1981;9:309–318.
5. Lewis RJ, Caccavale RJ, Sisler GE. Imaged thoracoscopic lung biopsy [see comments]. Chest 1992;102:60–62.

6. Lewis RJ, Caccavale RJ, Sisler GE, Mackenzie JW. Video-assisted thoracic surgical resection of malignant lung tumors. J Thorac Cardiovasc Surg 1992;104:1679–1685.
7. Lewis RJ, Caccavale RJ, Sisler GE, Mackenzie JW. One hundred consecutive patients undergoing video-assisted thoracic operations. Ann Thorac Surg 1992;54:421–426.
8. Landreneau RJ, Dowling RD, Ferson PF. Thoracoscopic resection of a posterior mediastinal neurogenic tumor. Chest 1992;102:1288–1290.
9. Landreneau RJ, Mack MJ, Hazelrigg SR, et al. Video-assisted thoracic surgery: basic technical concepts and intercostal approach strategies. Ann Thorac Surg 1992;54:800–807.
10. Dowling RD, Ferson PF, Landreneau RJ. Thoracoscopic resection of pulmonary metastases. Chest 1992;102:1450–1454.
11. Dowling RD, Landreneau RJ, Wachs ME, Ferson PF. Thoracoscopic Nd:YAG laser resection of a solitary pulmonary nodule. Chest 1992;102:1903–1905.
12. Bensard DD, McIntyre RJ, Waring BJ, Simon JS. Comparison of video thoracoscopic lung biopsy to open lung biopsy in the diagnosis of interstitial lung disease. Chest 1993;103:765–770.
13. Loddenkemper R, Boutin C. Thoracoscopy: present diagnostic and therapeutic indications. Eur Respir J 1993;6:1544–1555.
14. Roviaro G, Rebuffat C, Varoli F, Vergani C, Mariani C, Maciocco M. Videoendoscopic pulmonary lobectomy for cancer. Surg Laparosc Endosc 1992;2:244–247.
15. Roviaro G, Varoli F, Rebuffat C et al. Major pulmonary resections: pneumonectomies and lobectomies. Ann Thorac Surg 1993;56:779–783.
16. Dajczman E, Gordon A, Kreisman H, Wolkove N. Long-term post-thoracotomy pain [see comments]. Chest 1991;99:270–274.
17. Stanley DG. Thoracoscopic lobectomy. J Tenn Med Assoc 1992;85:463–464.
18. Kirby TJ, Rice TW. Thoracoscopic lobectomy. Ann Thorac Surg 1993;56:784–786.
19. Kirby TJ, Mack MJ, Landreneau RJ, Rice TW. Initial experience with video-assisted thoracoscopic lobectomy. Ann Thorac Surg 1993;56:1248–1252.
20. Walker WS, Carnochan FM, Pugh GC. Thoracoscopic pulmonary lobectomy. Early operative experience and preliminary clinical results. J Thorac Cardiovasc Surg 1993;106:1111–1117.
21. Walker WS, Carnochan FM, Tin M. Thoracoscopy assisted pulmonary lobectomy. Thorax 1993;48:921–924.
22. Hazelrigg SR, Nunchuck SK, LoCicero J. Video Assisted Thoracic Surgery Study Group data. Ann Thorac Surg 1993;56:1039–1043.
23. McKenna RJ. Lobectomy by video-assisted thoracic surgery with mediastinal node sampling for lung cancer. J Thorac Cardiovasc Surg 1994;107:879–881.
24. Ginsberg RJ. Thoracoscopy: a cautionary note. Ann Thorac Surg 1993;56:801–803.
25. Mack MJ, Gordon MJ, Postma TW, et al. Percutaneous localization of pulmonary nodules for thoracoscopic lung resection. Ann Thorac Surg 1992;53:1123–1124.
26. Mack MJ, Shennib H, Landreneau RJ, Hazelrigg SR. Techniques for localization of pulmonary nodules for thoracoscopic resection. J Thorac Cardiovasc Surg 1993;106:550–553.

